# Evidence for conservation and selection of upstream open reading frames suggests probable encoding of bioactive peptides

**DOI:** 10.1186/1471-2164-7-16

**Published:** 2006-01-26

**Authors:** Mark L Crowe, Xue-Qing Wang, Joseph A Rothnagel

**Affiliations:** 1 The Australian Research Council Special Research Centre for Functional and Applied Genomics, The University of Queensland, Brisbane, Queensland 4072, Australia; 2 Institute for Molecular Bioscience, The University of Queensland, Brisbane, Queensland 4072, Australia; 3 School of Molecular and Microbial Sciences, The University of Queensland, Brisbane, Queensland 4072, Australia

## Abstract

**Background:**

Approximately 40% of mammalian mRNA sequences contain AUG trinucleotides upstream of the main coding sequence, with a quarter of these AUGs demarcating open reading frames of 20 or more codons. In order to investigate whether these open reading frames may encode functional peptides, we have carried out a comparative genomic analysis of human and mouse mRNA 'untranslated regions' using sequences from the RefSeq mRNA sequence database.

**Results:**

We have identified over 200 upstream open reading frames which are strongly conserved between the human and mouse genomes. Consensus sequences associated with efficient initiation of translation are overrepresented at the AUG trinucleotides of these upstream open reading frames, while comparative analysis of their DNA and putative peptide sequences shows evidence of purifying selection.

**Conclusion:**

The occurrence of a large number of conserved upstream open reading frames, in association with features consistent with protein translation, strongly suggests evolutionary maintenance of the coding sequence and indicates probable functional expression of the peptides encoded within these upstream open reading frames.

## Background

The 5' untranslated regions (5' UTRs) of vertebrate mRNAs typically vary from a few tens of bases up to several hundred bases in length, and contain a variety of features which affect the efficiency of translation of the main coding sequence (CDS) of the transcript. These include the length and secondary structure of the 5' UTR, the sequence context of the initiation codon of the main CDS and the presence of upstream AUG codons (uAUGs). The original scanning model of mRNA translation (reviewed in [1]) postulates that the translating ribosome enters the mRNA at the 5' end and processes linearly down the molecule until reaching the first AUG, at which point translation is initiated. Further work has since resulted in refinements to this model, such as the recognition of leaky scanning, where uAUGs in a sequence context which varies from the optimal consensus for initiation of translation are bypassed by the ribosome complex, and of reinitiation, where the ribosome resumes scanning after translation of upstream open reading frames (uORFs) (reviewed in [2]).

Both the leaky scanning and ribosome reinitiation mechanisms typically result in a decrease in efficiency of translation from subsequent initiation codons, in some cases virtually eliminating translation of the main CDS, although more usually causing between two and 50-fold reduction in protein levels [3-8]. It has therefore been proposed that the presence of uAUGs may represent a method of post-transcriptional regulation, through repression of translation from the main AUG. This theory is supported by the identification of transcriptional and splice variants with an identical main CDS but with 5' UTRs containing varying numbers of uAUGs [6,9,10], as well as cases of an uAUG repressing translation in one tissue type while not affecting it in another [11,12].

Furthermore a number of diseases have been associated with uAUGs. These are caused either through mutations introducing or eliminating uAUGs, resulting in a consequent decrease or increase in translation efficiency [13-16], or by physiological changes which may affect the degree of repression by a particular uAUG [17] or may change splicing patterns of the 5' UTR to alter the number of uAUGs [6].

In addition to repression of translation by uAUGs, additional regulation of mRNAs containing uORFs can be brought about by the uORF affecting transcript stability. The premature termination codon model for nonsense-mediated decay (NMD) of mRNA (reviewed in [18-20]) suggests that transcripts containing uORFs would effectively have a premature termination codon at the end of the uORF. Translation of the uORF would reduce progression of the ribosome to the main CDS and ultimately to its termination codon, and therefore may result in increased targetting of these molecules for NMD, thereby reducing the steady-state level of that particular transcript. Some uORFs do appear to have such a role in destabilization of their mRNA by promoting NMD [21-23] while others have been shown to similarly destabilize mRNA, but via an NMD-independent pathway [24].

Despite the examples above, most studies on the effects of uAUGs have considered the role of the uAUG as acting solely as an alternative site of ribosome initiation and therefore to repress translation of the main CDS; the role of the associated uORF is apparently considered largely incidental. Even in reports describing the function of the uORF itself, the role of the translated peptide is primarily a cis-acting one, either causing ribosome stalling at the termination codon of the uORF and a high level of repression of the main CDS [25,26] or, more occasionally, affecting mRNA stability. In this report we describe our identification of a substantial number of uORF-encoded peptides (uPEPs) which are highly evolutionarily conserved and which we propose have functions beyond that of simply reducing translation of the main CDS.

## Results

### Presence of uAUGs and uORFs

For this experiment, we restricted our analysis to uORFs of 20 to 99 codons in length. While these specific values are somewhat arbitrary, we chose them to maximise the probability that the uORFs chosen for further analysis were genuine and not affected by sequencing errors or cloning artefacts. While it is possible that a uORF of less than 20 codons could encode a functional peptide, ORFs longer than this are potentially easier to identify as conserved between species and furthermore would be more amenable to subsequent experimental validation. We chose the upper limit after our initial results indicated that only a small fraction of uORFs (~5%) were more than 100 codons long, so their exclusion was unlikely to greatly bias the analysis, while their inclusion would lead to the potential for contamination of our results by artefacts such as co-ligated cDNA clones or misidentification of the main CDS in a cDNA clone.

In the RefSeq release 6 database, there are 21768 human and 17106 mouse mature mRNA sequences. Of these, approximately 75% and 66% respectively have an annotated 5' UTR of 60 bases or more in length (exact figures given in table 1). The difference in the proportion of long 5' UTRs is primarily because of a much higher number of mouse sequences containing only the main CDS, with no 5' UTR given (11% of mouse sequences, compared with only 4.5% of human ones). Of the population of mRNAs with long 5' UTRs (>60 bases), approximately 55% have at least one uAUG, with about 25% having one or more uORFs (average about 1.9 uORFs). We only considered uORFs which had a stop codon either within the 5' UTR or no more than 30 bases (ten codons) into the main CDS since uORFs that extend into the main coding region may potentially be conserved as a side-effect of selection of the main open reading frame, rather than by any specific selection on their own peptide products. By limiting the reading frame overlap to less than half of the minimum length of the uORFs being investigated in this study, we hoped to minimise any biasing effect caused by this mechanism.

The human dataset has approximately 15% redundancy among uORF sequences, in contrast to only around 1% in the mouse; this may be a result of a lack of depth of the mouse mRNA resource, since these duplicates seem to mostly be the result of splice variants with similar or identical 5' UTRs. This is supported by the redundancy of gene name annotation of the two datasets, which is 3.7% for mouse but 16.9% for the human sequences.

Our final filter was to remove any uPEPs with homology to known proteins, since our preliminary results had indicated that a considerable number of RefSeq entries appeared to have frameshift mutations or introduced stop codons within the main CDS, which had led to internal ATG codons being described as the start of the CDS. This results in a 5' truncation of the annotated main CDS, the starting section of which will then appear to form a uORF in the 5' UTR. By using a relatively stringent filter, we removed approximately 10% of the uPEPs which showed more than a small degree of similarity to proteins in the NCBI non-redundant peptide sequence database, thus minimising the possibility that the uORFs are in fact part of a main CDS, and appear to be in the 5' UTR as a consequence of incorrect annotation. This filter also resulted in only novel sequences being further characterised. Of course, it is possible that uPEPs resulting from such sequencing and annotation errors would still pass our filtering process if they represented previously unidentified variants of the main reading frame, but in view of the depth of the existing protein databases, we believe that this would affect relatively few sequences.

An equivalent filtering process was applied to ORFs identified in the 3' UTRs. Since 3' UTRs are typically longer than 5' ones, we found approximately 30% more 3' UTRs that were longer than 60 bases, totalling four times more sequence data than the 5' UTR set. These 3' UTRs contained ten times as many AUGs and ORFs as were observed in 5' UTRs. However the proportions of downstream open reading frames (dORFs) which were filtered out because of duplication or matching to an entry in the nr protein database were similar to those of uORFs. The results of the full filtering process are summarised in tables 1 and 2 (for 5' UTRs and 3' UTRs respectively).

Although 40–50% of mRNAs have at least one uAUG codon, this percentage is in fact much lower than would be expected either by chance or by comparison with the frequency of other upstream codons (~90% of mRNAs in this dataset), supporting the theory that there is significant selection against AUG codons in 5' UTRs [27,28]. In contrast, the 3' regions of the mRNAs which we have tested contain AUGs at approximately the level expected by chance based on the sequence composition and length. This absence of selection against downstream AUG codons, in combination with the generally accepted view that ORFs in the 3' UTR are not translated, supports our use of the 3' UTR as a valid negative control data set.

### Conservation between species

From the 6454 human and 5089 mouse uPEPs which passed all our filtering processes, we identified 204 homologous pairs (defining homologs as uPEPs with a blastp E value of < 0.01, identity of > 50%, aligned start and stop codons and no insertions or deletions) [see Additional files 1, 2]. The proportion of uPEPs with homologs was therefore approximately 3.5%, in comparison to less than 1% of peptide translations of dORFs under the same conditions (500 pairs from 65258 human and 52786 mouse dORFs), showing that uPEPs are more highly conserved than non-translated downstream sequences.

A HomoloGene cluster was identified for both the human and mouse transcript sequences for 177 of the uPEP pairs. In 169 of these cases (95%), both transcripts mapped to the same cluster; therefore the vast majority of the identified pairs appear to be genuine orthologs. This was also the case for the downstream pairs, of which just less than 90% are orthologous.

We identified 79 human/mouse homolog uORF pairs where both human and mouse sequences matched entries in the EST database with an aligned start codon. In total these matched 241 unique EST sequences, mostly from cow (*Bos taurus*) and pig (*Sus scrofa*), of which 201 (82%) also contained a stop codon aligned with that of the mouse and human uORFs. Of the 40 without an aligned stop codon, 15 had one at an adjacent codon, and a further 16 had a stop codon within 5 codons of the human homolog (compared with ~4 and ~7 which would be expected if stop codons were randomly distributed within this region of the 5' UTR). This overrepresentation of in-frame stop codons is a consistent with there being selective pressure to maintain the length of these uORFs. In contrast, 72% of the ESTs matching to dORFs had an aligned stop codon, while of the remainder, the observed number having a stop codon within five codons (155) was almost exactly that predicted by random assignment of stop codons (154).

### Strength of AUG context

The efficiency of initiation of translation from a given AUG codon is determined in part by the local sequence context. The most efficient context is known as the Kozak sequence (GCC^A^/_G_CCAUGG), and two positions within this sequence, -3 and +4 (the A of the AUG codon is designated +1) are the most critical for determining the strength of the initiator and hence translation efficiency [29,30]. AUGs matching the consensus at both of these sites are described as being in an optimal context, those matching at one are strong, and those matching neither are described as weak.

The proportions of AUG in each context are given in table 3 (expressed as a percentage of all AUGs in that category). Among the conserved uORFs, the proportion with an optimal AUG context is almost double that observed for other classes of uORF (figure 2). While this is still lower than is observed for the main coding region AUGs, it is a strong trend and one not observed in the downstream controls, where conserved ORFs have no noticeable enrichment for optimal AUG context.

Interestingly the proportion of optimal AUGs among the main CDSs of mRNAs with one or more uORFs is substantially lower than for mRNAs without a uORF (33.2% c.f. 41.3%). This is not the case for dORFs, where the proportion of optimal and strong main AUGs is unaffected by the presence or absence of a dORF.

### Synonymous mutation rates

We joined each of the 204 human and mouse DNA uORF sequences sequentially to create two aligned composite sequences. This alignment of 17766 bases was used to calculate a ratio of synonymous to non-synonymous nucleotide substitutions using the SNAP.pl program [31-33]. This ratio was 1.65, while the artificially-generated control dataset gave a mean of 0.99 (ranging from 0.70 to 1.36 over 10,000 trials). The ratio of the adjacent control sequences was 1.12 for upstream and 1.11 for downstream sequence.

Insertion of one or two nucleotides at the beginning of each composite uORF sequence causes SNAP.pl to calculate the ratio for the two alternative reading frames. Synonymous mutations are mostly in the third nucleotide position of each codon, while substitution of either of the other two nucleotides generally causes non-synonymous changes. Consequently calculations using such frame-shifted sequences typically give lower ratios of synonymous to non-synonymous substitutions. This was the case for the composite uORF sequence, with ratios of 1.37 and 1.14 respectively in the +1 and +2 reading frames, and confirms that the relatively high proportion of synonymous substitutions is related to codon position and is not an artefact of nucleotide bias or specific mutation types.

Based on the distribution of the artificially-mutated control data set, the probability of achieving a synonymous to non-synonymous ratio of 1.65 with codon-neutral evolution is less than 10^-10^. This is true even assuming a background level of 1.12 (the observed value of the adjacent control sequence) rather than the theoretical 0.99. The composite ratio for dORFs produced a ratio of 1.29, still significantly above the expected value for neutral mutation but at a much lower level than the uORFs (p < 0.01). This value also showed a much smaller change for the two alternate reading frames (1.22 for both), suggesting that it may be a consequence of the sequence composition and not codon dependent.

An alternative explanation for the high ratio value of the dORF sequences is that it is an artefact of the selection procedures used, namely that of choosing ORFs conserved at a peptide level. In this case, it provides a reference of the background level from which the uORF composite sequence is still highly significantly different (p < 10^-5^).

For this analysis of synonymous and non-synonymous changes, we used a single composite sequence from each organism rather than performing the analysis individually for each ORF. This allows us to compensate for the varying lengths of the ORFs while not affecting the overall number of synonymous and non-synonymous changes (because all the sequences remain in their original reading frame). Furthermore, since many of the ORF sequences are short (282 sequences are shorter than 100 nucleotides) and have relatively few changes, a single mutation can have a disproportionately large effect if testing the sequences individually; the use of a single long sequence reduces the impact of these outliers.

## Discussion

It is clear from our analyses and those of other studies [27,28,34-36] that a large proportion of mRNAs, probably between 40% and 50%, contain at least one upstream AUG codon, and that several thousand of these may potentially encode peptides of 20 or more amino acid residues. Indeed, in a recent study, Pesole and colleagues [27] found similar frequencies of uAUGs and uORFs to those reported here, using human, mouse and rat RefSeq mRNA sequences. However, probably due to differences in methodology, they identified fewer conserved uORFs, but they predicted that these would have biological activity.

To date the majority of characterised uORFs appear to function as cis-acting regulatory elements which inhibit initiation levels at the main AUG codon [3-8]. While this suggests that the phenomenon of uAUG-mediated repression is a common mechanism for post-translational regulation, we believe that the results of our identification and analysis of conserved uORFs indicates that a sub-category of uORFs may have more varied biological functions than simple repression of translation of a main coding sequence. We base this conclusion on our observations that uORFs conserved between human and mice show a range of features consistent with selection at the peptide level which are much less apparent among non-conserved uORFs.

Firstly, although the 3' UTR tends to be more conserved between species than the 5' region [37-39], we find nearly a four-fold higher proportion of human uORFs have a mouse ortholog than do dORFs. Secondly, the initial AUG codons of these conserved uORFs are much more likely to match both of the critical consensus sites for ribosome initiation than are non-conserved uORFs or control dORFs. The correlation between sequence conservation and selection for translation-enhancing AUG contexts in these specific ORFs is consistent with our hypothesis that they are likely to encode biologically relevant peptides. Thirdly, while most orthologous uORFs have both start and stop codons positionally conserved, those that do not, have a stop codon present within five codons and in frame nearly three times more frequently than would be expected by chance. This conservation in uORF length, which is not seen in the dORF control set, would be expected for short functional peptides, where even a few additional residues represents a proportionally large change in peptide size and structure. Finally there is a very significant mutational bias of uORFs in favour of synonymous substitutions, indicating selection at the peptide, rather than the nucleotide, level.

One argument against peptides encoded by uORFs having significant biological activity is the typical instability and rapid degradation of small peptides [8,40]. This argument maintains that uPEPs would not survive long enough to elicit measurable biological activity. In addition, uPEPs may not be synthesized at high enough levels to be functionally relevant, either because of low translation efficiencies or because their encoding uORFs are only present in rare transcript variants. However, translated uPEPs have been identified in human cells by mass spectrometry [41] and recently *trans *activity has been demonstrated for at least two small peptides in mammals, a 43 residue naturally occurring uPEP [42] and a recombinant 15 residue peptide [43]. Interestingly, in the former case, biological activity was found even though the peptide itself could not be detected. It is therefore clear that, although uORF-encoded peptides may exist at only very low levels within the cell, they still have the potential for *trans*-acting biological functions. These *trans*-acting functions remain to be elucidated, but uPEPs have the same characteristics as other intracellular peptides which have been predicted to regulate a host of protein-protein, RNA-protein and DNA-protein interactions [44].

In conclusion, we have carried out a genome-wide comparative analysis of human and mouse uORFs, and have identified a number of well conserved uORFs which show evidence of evolutionary selection at a peptide level. While previous analyses have implicated a small number of uPEPs in cis-acting post-transcription regulation, we believe that the presence of hundreds of uORFs, which are strongly conserved at the peptide level, suggests other biological roles for uPEPs.

## Methods

### Data sets

We used RefSeq release 6 human and mouse mRNA sequences from NCBI [45], taking only entries with an NM-prefixed accession number (i.e. those derived from mature mRNA sequences). We used the annotation provided within the GenBank format file to identify the start of the main CDS for each entry where this could be unambiguously identified; sequences where the CDS start point was not absolutely defined (i.e. anything not containing a line of the format 'CDS 123..567', where 123 and 567 are the start and end coordinates of the main CDS) were excluded from further analysis, as were sequences with short 5' UTRs (<60 bases). Based on the annotated CDS start points, we retrieved sequence from the beginning of the entries to 30 nucleotides past the annotated start of the main CDS.

For a control comparison, we also extracted 3' untranslated regions (3' UTRs) from the human and mouse RefSeq database. We identified stop coordinates of the main CDS as described above and, providing the 3' UTR was longer than 60 bases, we retrieved sequence from immediately after the stop codon to the end of the entry.

### Identification of uORFs

We screened the retrieved UTR sequences for the presence of ORFs of 20 to 99 codons in length starting with an AUG codon and terminating with a stop codon. Only uORFs with a stop codon within the 5' UTR or less than 30 bases into the main CDS were analysed further. Similarly, 3' control ORFs were required to have both a start and stop codon within the 3' UTR.

Although the RefSeq database is a non-redundant resource and contains no duplicate sequences, a considerable proportion of the ORFs which we identified were duplicated. This was mostly because more than one transcript variant shared the same ORF or ORFs. In these cases, we retained a single representative of each sequence, removing any other ORFs with an identical translated peptide sequence.

To minimise the possibility that the ORFs are in fact part of a main CDS, and appear to be in the UTR as a consequence of incorrect annotation, we performed a NCBI blastp alignment (E value cutoff of 0.01) with the peptide translation of all our identified ORF sequences against the NCBI nr protein database, and only continued analysis with ORFs which did not give any hits.

### Identification of conserved and orthologs ORFs

To identify ORFs conserved between human and mouse, we performed an NCBI blastp alignment of peptide translations of the mouse ORF sets against the equivalent human set using an E value cut off of 0.01, equivalent to that used in elimination of main coding sequence contamination. We screened matches identified from this search for ORFs where the initial methionine from both sequences was aligned, the peptides were the same length with homology extending along the whole length, and the overall identity between the two sequences was > 50%. Although aligned start codons and identical length are not necessarily features of conserved sequences, this requirement provides us with a subset of the most reliable homologous ORFs; it also simplifies the alignments required for calculation of synonymous and non-synonymous mutation rates.

We used the accession numbers of the original mRNA sequences for each of these human/mouse ORF pairs to search HomoloGene (NCBI). The pairs were considered to be orthologous if both mapped to the same HomoloGene identifier.

For each mouse/human ORF pair identified as described above, we used tblastn (peptide sequence against a translated DNA database) of peptide translations of both the mouse and human ORFs against the NCBI non-mouse, non-human EST database (E value cut off of 1.0) to identify potential uORF and dORF sequences from other species. Matches to rodent and primate species were discounted, as were matches where the initial AUG codon did not align with the reference sequence.

**Figure 1:**
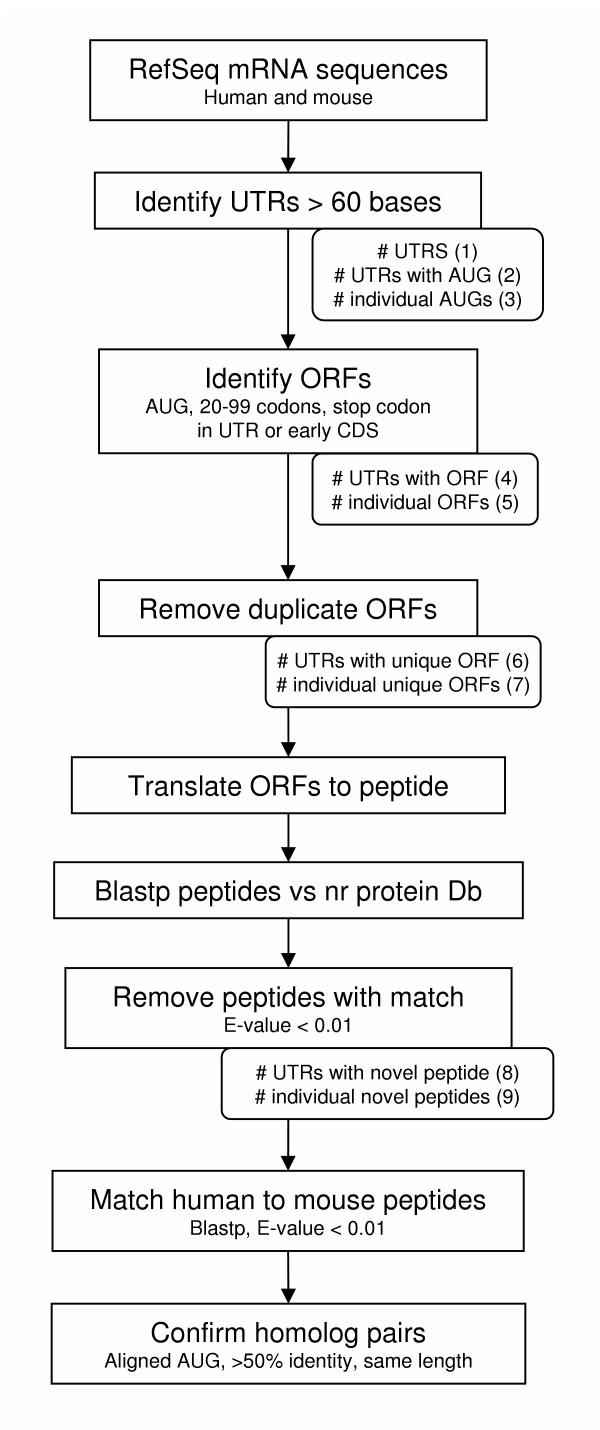
Flowchart of steps used in identification of conserved upstream and downstream ORFs. Numbers in parentheses indicate the corresponding count in tables 1 and 2.

**Figure 2:**
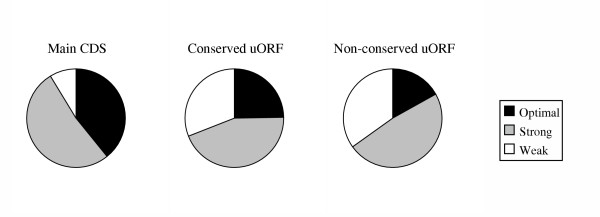
Proportion of AUG codons in optimal, strong or weak sequence contexts for main coding regions, interspecific conserved uORFs, and non-conserved uORFs.

A flowchart summarising the full screening process, from RefSeq datasets through to conserved ortholog pairs, is shown in figure 1.

### Strength of Kozak consensus

For all ORFs we evaluated the strength of the initiation codon based on the presence of specific nucleotides in the Kozak consensus sequence [29,30]. The two most critical nucleotide positions in determining the efficiency of initiation at a given AUG codon are A/G^-3 ^and G^+4 ^[29,30]. We therefore assigned an arbitrary 'strength score' to each AUG codon corresponding to how many of these critical nucleotides matched the consensus; either zero, one or two. We calculated the relative proportions of optimal (score of two), good (score of one) and weak (score of zero) for all ORF AUG and main AUG codons, as well as AUG codons of short ORFs (five codons or less) and theoretical predictions based on local nucleotide usage. Theoretical predictions were calculated as follows; optimal: ((p_G _+ p_A_) × (p_G_)), weak: ((p_C _+ p_T_) × (1 - p_G_)), good: (1 - (optimal + weak)), where p_N _was the proportion of that nucleotide in all long (>60 bases) UTRs from that species.

### Calculation of synonymous/non-synonymous mutation rates

To calculate an overall ratio of synonymous to non-synonymous changes across all conserved ORFs without biasing for sequence length, we created a single composite sequence for each species. We concatenated the total collection of conserved human and mouse ORFs, excluding the initial AUG and stop codon from each ORF, to generate two aligned composite ORFs. This alignment was analysed using the SNAP.pl program [31-33], which calculates the number of synonymous mutations as a proportion of possible synonymous mutations, and similarly for non-synonymous mutations, and returns a ratio of these two values. Deviations of this ratio away from one are indicative of positive or negative selective pressure on the peptides encoded by the DNA sequences.

We generated reference data by taking duplicate copies of the human composite sequence and computationally mutating the second copy at random using mutation rates of 5–20% (approximately that observed between the real sequences). The choice of replacement nucleotides was biased in accordance with the base content of the composite ORF. We used SNAP.pl to calculate the synonymous/non-synonymous mutation rate for the generated test sequences, and performed 10,000 repeats of this mutagenesis/ratio calculation approach to model the distribution of ratios generated by 'neutral' mutations.

To compensate for the possibility of our synonymous mutation rate calculations being affected by the overall level of sequence conservation between human and mouse, or by specific features of 5' and 3' UTR sequences, we used sequences immediately adjacent to, and of the same length as, the ORFs to generate additional control data. For the upstream ORFs, preceding sequence was retrieved, while for the downstream control ORFs, subsequent sequence was used (in this way, all control sequences were further away from the main coding region than the ORF, ensuring that none overlapped into this region). We created composite sequences for these controls and calculated synonymous/non-synonymous mutation rates as described above. We also calculated percentage sequence identities between the composite adjacent sequence alignments as well as between the composite ORF alignments.

## List of abbreviations

uAUG, upstream AUG codon; ORF, open reading frame; uORF, upstream open reading frame; dORF, downstream open reading frame; CDS, coding sequence; 5' UTR, five prime untranslated region; 3' UTR, three prime untranslated region; uPEP, upstream peptide.

## Authors' contributions

Author contributions: MLC, X-QW and JAR designed research; MLC and X-QW performed research; MLC analysed data; MLC, X-QW and JAR wrote the paper.

## Supplementary Material

Additional File 1 'Human/mouse homologous uORF pairs'. The conserved human uORFs with the corresponding mouse homolog. Identifers consist of the accession number of the parent RefSeq entry, with, where required, a subscript index to distinguish multiple uORFs from a single entry. Click here for file

Additional File 2 'uORF and uPEP sequences'. DNA and predicted protein sequences for each conserved human and mouse uORF together with the accession number of their parent RefSeq entry. N.B. DNA sequences do not include the stop codon. Click here for file

## Figures and Tables

**Table 1:** Frequency of upstream AUGs and ORFs.

	Human	Mouse
Number of mRNA sequences in initial dataset (1)	16504	11291
Number of mRNA sequences containing > 1 uAUG (2)	9531	6352
Total number of uAUGs (3)	35599	24308
Number of mRNA sequences containing > 1 uORF (4)	4557	2820
Total number of uORFs (5)	8216	5487
Number of mRNA sequences containing > 1 uORF after removal of duplicates (6)	3924	2795
Total number of uORFs after removal of duplicates (7)	7138	5430
Number of mRNA sequences containing > 1 uORF after removal of blast matches (8)	3650	2678
Total number of uORFs after removal of blast matches (9)	6454	5089

Frequency of upstream AUGs and ORFs in human and mouse RefSeq mRNA sequences. Initial dataset refers to all sequences of > 60 nucleotides annotated as 5' UTRs. Numbers in parentheses indicate the corresponding stage in the filtering flowchart (figure 1).

**Table 2:** Frequency of downstream AUGs and ORFs.

	Human	Mouse
Number of mRNA sequences in initial dataset (1)	21597	14790
Number of mRNA sequences containing > 1 dAUG (2)	19853	14327
Total number of dAUGs (3)	352301	229945
Number of mRNA sequences containing > 1 dORF (4)	16965	11598
Total number of dORFs (5)	85876	55815
Number of mRNA sequences containing > 1 dORF after removal of duplicates (6)	14252	11377
Total number of dORFs after removal of duplicates (7)	69202	54403
Number of mRNA sequences containing > 1 dORF after removal of blast matches (8)	13899	11299
Total number of dORFs after removal of blast matches (9)	65258	52786

Frequency of downstream AUGs and ORFs (dAUGs and dORFs) in human and mouse RefSeq mRNA sequences. Initial dataset refers to all sequences of > 60 nucleotides annotated as 3' UTRs. Numbers in parentheses indicate the corresponding stage in the filtering flowchart (figure 1).

**Table 3:** Comparison of the frequency of AUGs in different contexts.

Context of AUG present in:	Human			Mouse		
	Optimal	Strong	Weak	Optimal	Strong	Weak

All main CDS	39.8	51.4	8.8	38.7	52.6	8.6
Conserved uORFs	24.5	44.6	30.9	24.0	42.6	30.4
Non-conserved uORFs	16.5	48.8	34.7	17.1	47.5	35.3
Main CDS of uORF genes	33.6	52.4	13.9	32.7	53.7	13.6
Short uORFs	12.3	51.2	36.5	12.6	50.0	37.4
Theoretical uAUG*	15.4	50.4	34.2	14.8	50.2	34.9
Conserved dORFS	11.8	47.4	40.8	11.0	45.2	40.8
Non-conserved dORFs	11.4	49.0	39.6	12.0	48.0	40.1
Main CDS of dORF genes	39.2	51.6	9.2	38.7	52.7	8.5
Short dORFs	8.5	48.6	42.9	8.9	47.9	43.2
Theoretical dAUG*	10.6	49.7	39.7	10.6	49.6	39.6

Comparison of the frequency of AUGs in different contexts between uORFs and main ORFs. All values are expressed as percentages of the total number of AUGs in that class of ORF.

*Theoretical is defined as the predicted distribution of AUG contexts if the nucleotide selection at the two critical positions was randomly selected based on the sequence composition of all UTRs in that category (i.e. 5' UTR composition for upstream AUGs, 3' UTR composition for downstream AUGs).
